# Supplementary material for: Robust and distributed neural representation of action values
Source: eLife. 2021 Apr 20;10:e53045. doi: 10.7554/eLife.53045 (PMC8104958; doi:10.7554/eLife.53045)
Supplement: Supplementary file 1. — Top, statistical test results for 2θ plots. Orange shading, Y-component of the mean vector was tested for significant deviation from 0 (Wilcoxon rank-sum test, red indicates p-values <0.05). No shading, Y-component of the mean vector was compared across regions using one-way ANOVA (rat, F(7,2587) = 12.64, p=4.9 × 10^−16^; monkey, F(2,247) = 10.75, p=3.4 × 10^−5^) followed by Bonferroni post hoc tests. Significant differences (p-values <0.05) between regions are indicated in red. Bottom, statistical test results for 4θ plots. Orange shading, X-component of the mean vector was tested for significant deviation from 0 (Wilcoxon rank-sum test, red indicates p-values<0.05). No shading, X-component of the mean vector was compared across regions using one-way ANOVA (rat, F(7,2587) = 3.79, p=4.3 × 10^−4^; monkey, F(2,247) = 0.95, p=0.387) followed by Bonferroni post hoc tests. Significant differences (p-values <0.05) between regions are indicated in red. [file elife-53045-supp1.docx]

| ***2θ*** | | | | | | | | |
| --- | --- | --- | --- | --- | --- | --- | --- | --- |
| **Rat** | |  |  |  | **Monkey** | | | |
| **DMS** | 3.0×10^-6^ |  |  |  | **CD** | **VS** | **DLPFC** | **Areas** |
| **DLS** | 1 | 1.3×10^-5^ |  |  | 0.098 | 3.8×10^-4^ | 1 | **CD** |
| **VS** | 0.014 | 0.003 | 2.1×10^-6^ |  |  | 0.010 | 7.7×10^-5^ | **VS** |
| **OFC** | 0.018 | 0.005 | 1 | 3.2×10^-72^ |  |  | 0.001 | **DLPFC** |
| **mPFC** | 0.382 | 0.078 | 0.912 | 1 | 8.8×10^-31^ |  |  |  |
| **ACC** | 0.403 | 0.083 | 0.942 | 1 | 1 | 7.5×10^-27^ |  |  |
| **M2** | 0.130 | 1 | 6.7×10^-7^ | 1.6×10^-13^ | 1.0×10^-8^ | 1.6×10^-8^ | 0.003 |  |
| **CA1** | 1 | 1 | 0.020 | 0.013 | 0.530 | 0.565 | 0.007 | 9.0×10^-9^ |
| **Areas** | **DMS** | **DLS** | **VS** | **OFC** | **mPFC** | **ACC** | **M2** | **CA1** |

| ***4θ*** | | | | | | | | |
| --- | --- | --- | --- | --- | --- | --- | --- | --- |
| **Rat** | |  |  |  | **Monkey** | | | |
| **DMS** | 0.455 |  |  |  | **CD** | **VS** | **DLPFC** | **Areas** |
| **DLS** | 1 | 0.410 |  |  | 0.098 | 1 | 0.614 | **CD** |
| **VS** | 0.356 | 0.782 | 0.027 |  |  | 0.109 | 0.952 | **VS** |
| **OFC** | 0.148 | 0.984 | 1 | 2.5×10^-5^ |  |  | 0.03 | **DLPFC** |
| **mPFC** | 0.036 | 0.319 | 1 | 1 | 1.2×10^-6^ |  |  |  |
| **ACC** | 0.413 | 1 | 1 | 1 | 1 | 1.3×10^-4^ |  |  |
| **M2** | 1 | 1 | 0.613 | 0.224 | 0.051 | 0.732 | 0.029 |  |
| **CA1** | 1 | 1 | 1 | 0.654 | 0.156 | 1 | 1 | 0.332 |
| **Areas** | **DMS** | **DLS** | **VS** | **OFC** | **mPFC** | **ACC** | **M2** | **CA1** |

**Supplementary file 1. Statistical test results for 2θ and 4θ plots. Top**, statistical test results for 2θ plots. Orange shading, Y-component of the mean vector was tested for significant deviation from 0 (Wilcoxon rank-sum test, red indicates *p-*values < 0.05). No shading, Y-component of the mean vector was compared across regions using one-way ANOVA (rat, F(7,2587) = 12.64, *p* = 4.9×10^-16^; monkey, F(2,247) = 10.75, *p* = 3.4×10^-5^) followed by Bonferroni post-hoc tests. Significant differences (*p*-values < 0.05) between regions are indicated in red. **Bottom**, statistical test results for 4θ plots. Orange shading, X-component of the mean vector was tested for significant deviation from 0 (Wilcoxon rank-sum test, red indicates *p-*values < 0.05). No shading, X-component of the mean vector was compared across regions using one-way ANOVA (rat, F(7,2587) = 3.79, *p* = 4.3×10^-4^; monkey, F(2,247) = 0.95, *p* = 0.387) followed by Bonferroni post-hoc tests. Significant differences (*p*-values < 0.05) between regions are indicated in red.
